# Changing Patterns of Dengue Epidemiology and Implications for Clinical Management and Vaccines

**DOI:** 10.1371/journal.pmed.1000129

**Published:** 2009-09-01

**Authors:** Cameron P. Simmons, Jeremy Farrar

**Affiliations:** Hospital for Tropical Diseases, Wellcome Trust Major Overseas Programme, Oxford University Clinical Research Unit, Ho Chi Minh City, Viet Nam

## Abstract

Cameron Simmons and Jeremy Farrar discuss the changing patterns of dengue epidemiology and the implications for clinical management and vaccines, drawing upon a recent study by Derek Cummings and colleagues in Thailand.

**Linked Research Article**

This Perspective discusses the following new study published in *PLoS Medicine*:

Cummings DAT, Iamsirithaworn S, Lessler JT, McDermott A, Prasanthong R, et al. (2009) The impact of the demographic transition on dengue in Thailand. PLoS Med 6(9): e1000139. doi:10.1371/journal.pmed.1000139

Analyzing data from Thailand's 72 provinces, Derek Cummings and colleagues find that decreases in birth and death rates can explain the shift in age distribution of dengue hemorrhagic fever.

## Background

Dengue is the most important arboviral disease of humans and has emerged as a global public health problem. The dengue case burden, and the number of countries reporting outbreaks, has increased 10-fold in the last 30 years. The rapidly urbanising developing and middle-income countries of Asia and the Americas are most affected. Disease prevention relies, mostly unsuccessfully, on control of the principal mosquito vector, *Aedes aegypti*. Whilst there are currently no licensed vaccines for dengue, promising candidates are progressing through clinical development pipelines, and the prospect of a dengue vaccine becoming available in the next 10 years is a real one [1].

The four dengue virus serotypes co-circulate in many endemic areas, and intriguingly, a second infection caused by a serotype different from an individual's first exposure is associated with greater risk for severe disease. Oscillations in severe dengue incidence have been described in Thailand over the last 30 years, with peaks in incidence and serotype dominance occurring on regular cycles [2]. Interestingly, Viet Nam does not necessarily follow the same cyclical pattern [3].

## The Changing Dynamics of Dengue in Thailand

Severe dengue remains primarily a disease of children in endemic areas, but a measurable increase in the median age of cases has been documented in Thailand and anecdotally observed across much of Asia. In this issue of *PLoS Medicine*, Derek Cummings and colleagues [4] have shed light on a plausible mechanism for this increase by examining temporal changes in the demographics of the Thai population since 1985 and relating these changes to the incidence of severe disease. During this period, the median age of cases in Thailand rose from ∼9 years of age to ∼17 years of age. Even more dramatic, between 1999 and 2005 the median age of cases rose from ∼13 years of age to ∼17 years of age. Such changes in the age-related case burden of dengue have been attributed to reductions in the force of infection (the rate at which susceptible individuals become infected) due to socioeconomic developments and/or vector control that place fewer people at risk of exposure [5]. However, socioeconomic developments in Thailand have also been accompanied by changes in the population structure, with a lower birth rate in 2000 compared to 1985 and therefore an increase in the median age of the population over this period. We have observed equivalent changes to the population structure in Viet Nam over the same time interval. By multivariate analysis, Cummings et al. demonstrated that an increase in median age and in the proportion of homes constructed with permanent materials were independently associated with the reduction in the mean force of infection in the 72 provinces of Thailand. Increasing median age, but not other variables, was also independently associated with an increase in the period, or time in years, between dengue epidemic peaks. Sensibly, Cummings et al. have recognised that other factors such as young, non-immune adults migrating from lower transmission rural settings to seek employment in urban locations with higher levels of transmission could also explain the change in age-related dengue epidemiology.

## What Are the Implications for Vaccines and Clinical Practice?

The estimated reduction in the force of infection in Thailand makes only a modest impact on the proportion of individuals that would need to be effectively vaccinated to stop disease transmission, with Cummings et al. estimating the critical vaccination threshold dropping from 85% to 80%. More importantly, the targeted age group for vaccination should now be reconsidered in light of a higher average age (∼16 years) of all reported dengue cases in Thailand. Thus, vaccination of children as they enter primary school, rather than during infancy, might be a more rational and cost-effective approach to disease prevention, particularly for vaccines that might not elicit lifelong immunity from the primary vaccination course and would therefore require booster doses at multi-year intervals. Indeed, if the mean age of dengue cases were to continue to rise, as is possible given the long-term trend in Thailand, then dengue would be predominantly a disease of young adults, and this would require a further rethinking of vaccination strategies.

A change in the age-burden of dengue also has implications for clinical practice. The management of severe dengue in children has been refined by clinical experience over the last 40 years and is focused on very careful replacement of the lost intravascular fluid. An increase in the number of adults in the case mix of dengue patients presents new challenges to clinicians as the clinical picture can differ from children [6]. Furthermore, the existing World Health Organization case classification scheme is based on clinical experience in children, and therefore refinements might be needed to encompass the spectrum of disease in adults [7]. A priority then is to understand if other dengue-endemic countries in Southeast Asia and Central and South America are experiencing similar temporal associations between demography and dengue epidemiology, and if so, why.
